# Changes in salivary electrolyte concentrations in mid‐distance trained sled dogs during 12 weeks of incremental conditioning

**DOI:** 10.14814/phy2.14493

**Published:** 2020-06-28

**Authors:** James R. Templeman, Noelle McCarthy, Michael I. Lindinger, Anna K. Shoveller

**Affiliations:** ^1^ Department of Animal Biosciences University of Guelph Guelph ON Canada; ^2^ The Nutraceutical Alliance Burlington ON Canada

**Keywords:** electrolyte minerals, exercise, saliva, sporting dogs

## Abstract

Regular exercise improves the health status of dogs; however, extreme exertion in the absence of adequate fluid and electrolyte replacement may negatively impact health and performance due to dehydration and cardiovascular stress. Unlike humans and horses, dogs thermoregulate predominantly through respiration and salivation, yet there is a dearth of literature defining exercise‐induced changes to canine salivary electrolytes. The study objective was to investigate the effects of exercise on salivary electrolyte concentrations, and to determine if adaptations may occur in response to incremental conditioning in client‐owned Siberian Huskies. Sixteen dogs were used, with an average age of 4.8 ± 2.5 years and body weight of 24.3 ± 4.3 kg. A 12‐week exercise regimen was designed to increase in distance each week, but weather played a role in setting the daily distance. Saliva samples were collected at weeks 0 (pre‐run, 5.7 km), 5 (pre‐run, 5.7, 39.0 km), and 11 (pre‐run, 5.7, 39.0 km). Samples were analyzed for sodium, chloride, potassium, calcium, magnesium, and phosphorous using photometric and indirect ion‐selective electrode analysis. When compared across weeks, sodium, chloride, potassium, and calcium concentrations did not differ at any sampling time point; however, phosphorus and magnesium concentrations increased from baseline. Data were then pooled across weeks to evaluate changes due to distance and level of conditioning. Sodium, chloride, and magnesium concentrations increased progressively with distance ran, suggesting that these electrolytes are primarily being lost as exercising dogs salivate. Repletion of these minerals may assist in preventing exercise‐induced electrolyte imbalance in physically active dogs.

## Graphical abstract

While salivation and respiration are the primary modes of thermoregulation in dogs, little is understood regarding salivary electrolyte profiles in exercising dogs. The goal of this project was to lay the groundwork necessary for future studies to build upon by investigating how short or extended bouts of exercise—as well as level of aerobic conditioning—might influence salivary electrolyte profiles in actively training sled dogs.

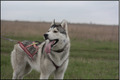

## 1 INTRODUCTION

Without adequate fluid and electrolyte replacement, exercise‐induced dehydration decreases athletic performance and negatively impacts health and wellbeing in human and canine athletes alike (Goulet, 2012; Young, Iacovino, Erve, Mosher, & Spector, 1959; Zanghi, Robbins, Ramos, & Otto, 2018). During prolonged exercise, evaporative cooling results in the loss of body water and cations (Hinchcliff, Reinhart, Burr, Schreier, & Swenson, 1997). In humans and horses, the primary method of heat dissipation as well as fluid and electrolyte losses occur through active sweating (Flouris & Schlader, 2015; McKeever, 2008). These losses cause decreases in total body water and plasma volume and, depending on the type of fluid loss (e.g., hypotonic, isotonic, or hypertonic), can increase plasma osmolarity. The reduced blood volume lessens cardiac filling and stroke volume while increasing heart rate, resulting in a degree of cardiovascular stress greater than that caused by exercise itself (Cheuvront, Carter, & Sawka, 2003). This may result in reduced blood flow to heat dissipation sites (e.g., skin, respiratory tract including mouth), resulting in inadequate heat transfer to the environment and excessive increases in body heat storage (Geor, McCutcheon, Ecker, & Lindinger, 2000). This will compromise the ability to dissipate heat, further compounding the issues related to dehydration (Von Duvillard, Braun, Markofski, Beneke, & Leithäuser, 2004). For example, in dogs induced with extracellular hyperosmolality—an outcome of hypertonic dehydration—internal temperature increased by nearly 2°C during 1 hr of submaximal exercise, an increase that was ~0.5°C greater than reported in the control dogs (Kozlowski, Greenleaf, Turlejska, & Nazar, 1980).

Unlike humans and horses though, dogs primarily thermoregulate by way of convection and radiation during thermal panting, as well as by conduction through the skin; though, the thermoregulatory role that conductive cooling plays for dogs is relatively minor, particularly when ambient temperatures are below 30°C (Hammel, Wyndham, & Hardy, 1958). Thus, dogs likely lose electrolytes by way of salivation (Blatt, Taylor, & Habal, 1972; Ermon, Yazwinski, Milizio, & Wakshlag, 2014; Villiger et al., 2018); however, fluid losses that occur during thermal panting are considered “insensible” and consequently can be challenging to measure. It has been reported, though, that when dogs perform aerobic exercise in a state of hypertonic dehydration, they will preserve body water by greatly reducing the rate of fluid loss via salivary secretions (Baker, Doris, & Hawkins, 1983).

Exercise in the absence of adequate fluid and electrolyte replacement can result in hypertonic dehydration, where total body water (TBW) decreases while blood plasma osmolality and the concentration of sodium (Na) in the extracellular (interstitial and plasma) fluid increase. Measures of both blood plasma osmolality and plasma Na concentrations are regularly used as biomarkers for evaluating hydration status. Reports from human studies indicate that saliva osmolality also increases with progressive dehydration, similar to those changes seen in plasma; however, these changes may be influenced by the type of dehydration or fluid loss (Villiger et al., 2018). Comparatively, researchers cannulated the submaxillary gland of the dog and demonstrated that electrolyte minerals are concentrated into canine saliva in amounts approaching those found in normal blood plasma, as acinar cells of the submaxillary glands concentrate electrolytes (e.g., Na and potassium, K) directly from plasma into the saliva (Henriques, 1961). Furthermore, it has been hypothesized that for humans, changes in salivary electrolyte profiles, such as increased concentrations of Na and chloride (Cl), may be related to the level of work achieved during aerobic exercise (Chicharro et al., 1994). While these reports together may help to explain the link between exercise‐induced dehydration and changes in salivary electrolyte concentrations, there remains to be a dearth of literature reporting the effects of exercise on salivary electrolyte concentrations in dogs.

In order to better understand, and ultimately reduce the risks associated electrolyte imbalance in exercising dogs, it is essential to further our understanding of changes in salivary electrolyte concentrations. As such, the objective of this study was to evaluate exercise‐induced changes in canine salivary electrolyte concentrations, and to identify the electrolytes that are primarily lost in saliva during short and extended bouts of exercise. We hypothesized that salivary concentrations of Na and Cl would increase, and concentrations of select salivary K and calcium (Ca) would decrease in response to exercise. Additionally, we hypothesized that the concentrations of Na and Cl will continue to increase as the duration of exercise increases.

## 2 MATERIALS AND METHODS

### 2.1 Animals and housing

The present experiment was approved by the University of Guelph's Animal Care Committee (animal use protocol # 4008). Sixteen client‐owned domestic Siberian Huskies (nine females: four intact, five neutered; seven males: two intact, five neutered), with an average age of 4.8 ± 2.5 years and body weight (BW) of 24.3 ± 4.3 kg (mean ± standard error, SE), were used in the study. Dogs were housed and trained at an off‐site, privately owned facility (Rajenn Siberian Huskies, Ayr, ON) that had been visited and approved by the University of Guelph's Animal Care Services. During the study, dogs were pair or group‐housed in free‐run, outdoor kennels that ranged in size from 3.5 to 80 square meters and contained between 2 and 10 dogs each. Two dogs were removed from the trial (one on week 7, one on week 9) due to exercise‐related injuries; all data collected up until their respective points of removal were included in this report.

### 2.2 Diets and study design

For 2 weeks prior to the study period, all dogs were acclimated to a dry extruded kibble diet (Champion Petfoods LT.) that met or exceeded all National Research Council (2006) and Association of American Feed Control Officials (2016) nutrient recommendations and fed at intake levels predetermined from their historical feeding records. For additional information regarding the nutrient content of the diet as well as the ingredient deck, refer to Templeman et al. (2020). During the acclimation period and throughout the entire trial, dogs were fed once daily at 1700 hr. Initial BW were recorded at week ‐1 and thereafter, BW was measured weekly and food allotments were adjusted to maintain the dogs' week ‐1 BW. At feeding, all dogs were fed individually to allow adequate monitoring of food intake. Any orts were weighed and recorded daily. Throughout the study, all dogs were allowed ad libitum access to fresh water.

A 12‐week exercise regimen was proposed whereby exercise intensity and duration would increase incrementally. However, decisions made regarding the distance ran each day and number of stops (e.g., for water) were made with consideration of the ambient temperature and humidity. The average daily run distance (average of 4 days of running) for each week was as follows: 6.9, 12.9, 17.5, 23.8, 31.0, 37.2, 42.2, 30.0, 31.3, 34.5, 31.6, and 34.2 ± 4.4 km (mean ± SE) for weeks 0, 1, 2, 3, 4, 5, 6, 7, 8, 9, 10, and 11, respectively (Templeman et al., 2020). All training bouts commenced at approximately 08:00 hr. On the days of saliva collection, the exact distances ran were as follows: 5.7 km for week 0, 39.0 km (week 5), and 39.0 km (week 11). All saliva sampling days occurred on the same day (Monday, first training day of the week) for each week (0, 5, and 11). Training consisted of dogs running on a standard 16‐dog gangline. The gangline was attached to an all‐terrain vehicle with one rider who controlled the machine in its lowest gear. A pace of ~15 km/hr was maintained throughout the training period. Running pace and distance travelled was measured using a digital speedometer and odometer on the all‐terrain vehicle. Dogs were provided with water approximately every 10 km and were always provided with water immediately following a bout of exercise, or immediately following a post‐run saliva sampling. For additional details regarding the anticipated run distances for proposed incremental exercise regimen, refer to Templeman et al. (2020).

### 2.3 Sample collection and analysis

Using sterile gauze and forceps, saliva samples were collected from all dogs at week 0 (pre‐run, 5.7 km), week 5 (pre‐run, 5.7, 39.0 km), and week 11 (pre‐run, 5.7, 39.0 km). Samples were collected by rolling gauze around forceps and positioning the gauze under the dog's tongue and/or throughout the lining of the cheek within the buccal cavity for 30 s per sample (German, Hall, & Day, 1998). The gauze was then transferred to a sterile 50 ml centrifuge tube (Thermo Fisher Scientific) and samples were centrifuged at 3,500 *g* for 30 min at 4°C (Lavy, Goldberger, Friedman, & Steinberg, 2012; Tenovuo, Illukka, & Vähä‐Vahe, 2000) using a Beckman J6‐MI centrifuge (Beckman Coulter). Saliva was then collected, transferred into microcentrifuge tubes (Thermo Fisher Scientific), and frozen at −20°C prior to analysis (Lavy et al., 2012). At minimum, 0.3 ml of saliva was collected from each dog, at each sampling time point. Samples were analyzed for Na, Cl, K, Ca, magnesium (Mg), and phosphorous (P) by photometric and indirect ion‐selective electrode analyses using a Roche Cobas 6000 c501 biochemistry analyzer (Roche Diagnostics) at the Animal Health Laboratory (University of Guelph). Samples were analyzed in duplicate (100 µl of saliva per sample) with an intra‐assay coefficient of variation of <1.5% (range of linearity: Na, 20–180 mmol/L; Cl, 20–140 mmol/L; K, 3–100 mmol/L; P, 0.1–6.4 mmol/L; Mg, 0.1–2.0 mmol/L; Ca, 0.2–5.0 mmol/L).

An estimation of osmolarity was calculated according to Rasouli (2016) using the following equation: (number of) osmoles = n' × (number of) moles, in which the unit of n' is milliequvalent (mEq) per mmol, and n' is defined as the number of mEq of produced particles during solvation of 1 mmol of solute. Each of the electrolytes presented herein dissolve in water without ionization (n' = 1). Osmolarity was calculated based on the salivary electrolyte concentrations at each week, and at each sampling time point; however, it should be noted that this estimation is calculated without accounting for bicarbonate (HCO_3_) which, along with PvCO_2_, was reported to decrease in dogs subjected to short bouts of strenuous exercise (Robbins, Ramos, Zanghi, & Otto, 2017).

### 2.4 Statistical analysis

Data were analyzed using PROC MIXED of SAS (v.9.4; SAS Institute Inc.). Dog was treated as a random effect, and week and sampling time point were treated as fixed effects. Week was treated as a repeated measure and means were separated using the Tukey adjustment. The data were also pooled across weeks and analyzed using PROC MIXED of SAS with dog treated as a random effect and sampling time point treated as a fixed effect and as a repeated measure. Significance was declared at a *p ≤ *.05.

## 3 RESULTS

Pre‐run concentrations of P on week 5 were greater than weeks 0 or 11, and on week 11 were greater than week 0 (*p* ≤ .05; Table 1a). Concentrations of P at the 5.7‐km sampling time point were greater at weeks 5 and 11 compared to week 0 (*p* ≤ .05; Table 1b). Concentrations of Mg at 5.7 km were greater at week 11 than week 0 (*p* ≤ .05); however, levels at week 5 did not differ from either week 0 or 11 (*p* > .05; Table 1b). At the 39.0‐km sampling time point, Mg concentrations at week 11 were greater than week 5 (*p* ≤ .05; Table 1c). Salivary concentrations of Ca, Na, Cl, and K did not differ with week for any of the sampling time points (*p* > .05; Tables 1a–c).

**TABLE 1:** Mean electrolyte concentrations (±SE^d^) across weeks of training (0, 5, and 11) at the pre‐run (a), 5.7 km (b), and 39.0 km (c) sampling time points

	(a) Pre‐run sampling time point		
	Week 0 *n* = 16	Week 5 *n* = 16	Week 11 *n* = 14
Na, mmol/L^e^	81.81 ± 6.21	83.11 ± 6.21	82.59 ± 6.50
Cl, mmol/L	65.34 ± 8.52	73.29 ± 8.52	76.38 ± 9.02
K, mmol/L	30.97 ± 8.03	43.16 ± 8.03	37.47 ± 8.14
Ca, mmol/L	1.48 ± 0.13	1.68 ± 0.13	1.69 ± 0.14
Mg, mmol/L	0.71 ± 0.06	0.69 ± 0.06	0.56 ± 0.07
P, mmol/L	0.21 ± 0.03^c^	0.50 ± 0.03^a^	0.35 ± 0.04^b^

	(b) 5.7 km sampling time point		
	Week 0 *n* = 16	Week 5 *n* = 16	Week 11 *n* = 14
Na, mmol/L	98.69 ± 6.21	91.51 ± 6.21	94.88 ± 6.50
Cl, mmol/L	85.30 ± 8.52	70.53 ± 8.52	75.31 ± 9.04
K, mmol/L	29.18 ± 8.03	34.09 ± 8.03	36.19 ± 8.14
Ca, mmol/L	1.30 ± 0.13	1.15 ± 0.13	0.96 ± 0.14
Mg, mmol/L	0.71 ± 0.06^b^	0.88 ± 0.06^ab^	1.04 ± 0.07^a^
P, mmol/L	0.13 ± 0.03^b^	0.27 ± 0.03^a^	0.26 ± 0.04^a^

	(c) 39.0 km sampling time point		
	Week 0 *n* = 0	Week 5 *n* = 16	Week 11 *n* = 14
Na, mmol/L	—	101.75 ± 6.21	89.48 ± 6.50
Cl, mmol/L	—	92.84 ± 8.52	84.84 ± 9.04
K, mmol/L	—	36.37 ± 8.03	32.22 ± 8.14
Ca, mmol/L	—	1.09 ± 0.13	1.06 ± 0.14
Mg, mmol/L	—	1.15 ± 0.06^b^	1.46 ± 0.07^a^
P, mmol/L	—	0.28 ± 0.03	0.24 ± 0.04

^a,b,c^Values in a row with different superscript are different (*p* ≤ .05).

^d^ SE, standard error.

^e^ Na, sodium; Cl, chloride; K, potassium; Ca, calcium; Mg, magnesium; P, phosphorus.

Data were then pooled across week to evaluate changes due to run distance. Pre‐run P and Ca concentrations were greater than at 5.7 and 39.0 km (*p* ≤ .05), but concentrations at 5.7 and 39.0 km did not differ from each other (*p* > .05; Table 2). Magnesium concentrations at 39.0 km were greater than at 5.7 km, and at 5.7 km were greater than at the pre‐run sampling time point (*p* ≤ .05; Table 2). Pre‐run Na concentrations were lower than at 5.7 and 39.0 km (*p* ≤ .05), but concentrations at 5.7 and 39.0 km did not differ from each other (*p > *.05; Table 2). Chloride concentrations at 5.7 km were similar to pre‐run and 39.0 km; however, Cl concentrations at 39 km were greater than pre‐run concentrations (*p* ≤ .05; Table 2). Pooled K concentrations did not differ with any sampling time point (*p > *.05; Table 2).

**TABLE 2:** Mean electrolyte concentrations (±SE^d^) from data pooled across all weeks (0, 5, and 11) for each sampling time points (pre‐run, 5.7, 39.0 km)

	Pooled data from all weeks (0, 5, and 11)		
	Pre‐run *n* = 46	5.7 km *n* = 46	39.0 km *n* = 30
Na, mmol/L^e^	82.41 ± 5.05^b^	95.03 ± 5.05^a^	97.08 ± 5.90^a^
Cl, mmol/L	71.43 ± 7.21^b^	77.27 ± 7.21^ab^	89.71 ± 8.19^a^
K, mmol/L	37.20 ± 7.66	33.14 ± 7.66	31.88 ± 7.93
Ca, mmol/L	1.61 ± 0.09^a^	1.21 ± 0.09^b^	1.11 ± 0.12^b^
Mg, mmol/L	0.66 ± 0.04^c^	0.87 ± 0.04^b^	1.26 ± 0.06^a^
P, mmol/L	0.35 ± 0.02^a^	0.22 ± 0.02^b^	0.20 ± 0.03^b^

^a,b,c^Values in a row with different superscript are different (*p* ≤ .05).

^d^ SE, standard error.

^e^ Na, sodium; Cl, chloride; K, potassium; Ca, calcium; Mg, magnesium; P, phosphorus.

Saliva osmolarity was calculated using the combined mEq of Na, Cl, K, Mg, and P at each sampling time point for each week. No differences were observed within any sampling time point across any week (*p* > .05); however, differences were observed across sampling time point within week (Table 3). In week 0, estimated osmolarity at 5.7 km was significantly greater than the pre‐run time point with an increase of more than 15% (*p* ≤ .05; Table 3). At week 5, estimated osmolarity at did not differ between the pre‐run and 5.7‐km sampling time points (*p* > .05), but osmolarity at 39 km was greater than at both pre‐run and 5.7 km (*p* ≤ .05; Table 3) with increases of approximately 13.5% and 15%, respectively. Finally, by week 11, no differences in estimated osmolarity were observed across any sampling time point (*p* > .05).

**TABLE 3:** Mean osmolarity (±SE^d^) calculated for each sampling time point (pre‐run, 5.7, 39.0 km) across all weeks of training (0, 5, and 11)

	Osmolarity^e^ (mOsm/L)		
	Pre‐run	5.7 km	39.0 km
Week 0	180.53 ± 13.10^b^	215.50 ± 13.10^a^	—
Week 5	200.40 ± 13.10^b^	196.49 ± 13.10^b^	231.72 ± 13.78^a^
Week 11	196.47 ± 13.10	206.06 ± 13.10	207.24 ± 13.78

^a,b,c^Values in a row with different superscript are different (*p* ≤ .05).

^d^ SE, standard error.

^e^ Osmolarity calculated using the combined mEq of sodium, chloride, potassium, calcium, magnesium, and phosphorus at each sampling time point, across all weeks.

## 4 DISCUSSION

To the best of our knowledge, this is the first study to report salivary electrolyte concentrations in exercising dogs while investigating how the duration of exercise and physical conditioning affects these concentrations. The data presented herein indicate that when dogs participate in aerobic exercise, salivary concentrations of Na, Mg, and Cl increase, suggesting that these are the electrolyte minerals primarily lost in saliva during a bout of exercise. Moreover, electrolyte concentrations appear to change depending on the duration of a bout of exercise (e.g., Cl and Mg), suggesting that duration or intensity of aerobic exercise may influence changes in salivary electrolyte concentration. This indicates that electrolyte repletion may help prevent exercising dogs from entering states of electrolyte imbalance, especially dogs participating in extended and/or repetitive bouts of aerobic exercise.

Due to constraints related to the study design and in‐field sample collection, the effects of dehydration and changes in salivary production could not be directly measured; however, these pilot data will hopefully provide the groundwork necessary for future studies investigating the effects of exercise on canine salivary electrolytes. The calculated osmolarity data (Table 3) indicate that the level of pre‐exercise hydration for these dogs did not differ throughout the 12‐week trial period, but that signs of hypertonic dehydration were present at the end of the runs in week 0 and week 5. Of interest is a possible adaptive response to conditioning, as calculated osmolarity was elevated at 5.7 km in week 0, but not in subsequent weeks, and calculated osmolarity was then elevated at 39 km in week 5, but not subsequently in week 11. While the mechanism(s) underlying these reductions in salivary osmolarity remain unknown, the evidence suggests that the dogs had adapted to the short bout of exercise within 5 weeks, and to the extended bout of exercise within 11 weeks. These data provide evidence of dehydration in exercising dogs without having measured changes in body mass, and also indicate that as dogs become physically conditioned, they appear to employ adaptive measures to reduce the severity of hypertonic dehydration induced by either short or extended bouts of aerobic exercise.

In humans subjected to increasing levels of aerobic exercise, secretion of certain salivary electrolytes display a biphasic response. At low‐to‐moderate levels of exercise, concentrations of Na, Cl, and K remain relatively unchanged (comparable to what was reported in other studies: Dawes, 1981; Rutherfurd‐Markwick, Starck, Dulson, & Ali, Starck, Dulson & Ali, 2017; Shannon, 1967); however, once a certain work rate is achieved, Na and Cl concentrations in saliva increase dramatically while K remains stable (Chicharro et al., 1994). A similar pattern was observed in the current study, as Na and Cl concentrations increased during exercise while K concentrations stayed constant. As well, the Cl concentration increased only once the dogs were subjected to an extended bout of exercise, suggesting that, as with humans, salivary Cl concentrations may also follow a biphasic response depending on the work rate. This may also indicate that the work rates reached in previous studies with humans were simply not high enough to elicit a change in salivary electrolyte concentrations. Convertino, Keil, Bernauer, and Greenleaf (1981) reported that a minimum intensity of 40% VO_2_ max was required to elicit a change in plasma osmolality in humans, supporting the potential of a relationship between electrolytic shifts and the intensity of work performed. Or perhaps, this biphasic response of salivary electrolytes to level of work may have merely been a function of the study subjects achieving differing degrees or types (hypotonic, isotonic, hypertonic) of dehydration. While dehydration was not analytically evaluated in either the current study or the work by (Chicharro et al. (1994), based on calculated osmolarity in the current study, it appears as though this response (or perhaps, degree/type of dehydration) may be influenced not only by duration of exercise (e.g., work rate), but also by degree of aerobic conditioning. At baseline, a significant increase in salivary osmolarity was observed with only a short bout of exercise; however, after the dogs were subjected to 5 weeks of aerobic conditioning, equivalent changes in osmolarity were only evident following an extended bout of exercise. Moreover, as the dogs progressed further into the conditioning regimen (by week 11), no changes in salivary osmolarity were observed after either short or extended bouts of exercise. In the future, a comparative analysis of salivary electrolyte concentrations and osmolarity in dogs subjected to varying durations of aerobic exercise over an extended conditioning period is warranted to confirm whether duration of a single bout of exercise, or degree of aerobic conditioning affects the type or degree of dehydration that is occurring, and to identify the influence those changes may have on salivary electrolyte profiles.

In exercising horses exposed to cool and dry ambient conditions, similar patterns were reported to that of Chicharro et al. (1994), with sweat osmolality, as well as Na and Cl concentrations increasing as a bout of exercise persisted (McCutcheon, Geor, Hare, Ecker, & Lindinger, 1995). The exercise induced increases in sweat osmolality were paralleled by an increase in sweating rate (McCutcheon et al., 1995), and a similar relationship has been identified between saliva flow rate and salivary electrolytes in humans. Using stimulated parotid saliva, Na and Cl concentrations have been reported to increase with saliva flow rate while K was unaffected (Asking & Emmelin, 1985; Henriques, 1961; Thaysen, Thorn, & Schwartz, 1954). Recent studies in humans have demonstrated that sympathetic stimulation during submaximal exercise may actually decrease saliva flow rate; however, this appears to occur simultaneously with an increase in the concentration of some salivary electrolytes such as Na and Mg (Chicharro et al., 1999). Though, it should be noted that the sympathetic response seen in humans may vary from those of dogs due to considerable differences in their cardiovascular capacity (Poole & Erickson, 2011). In order to better understand the mechanisms behind these exercise duration‐induced changes in electrolyte concentration, future studies should attempt to evaluate salivary flow rate as well as salivary electrolyte concentrations under similar conditions in exercising dogs.

The compartmentalization of electrolytes may also, in part, explain the differences observed in salivary concentration depending on duration of exercise in the current study. Sodium and Cl are primarily extracellular ions, while Mg, Ca, and K are largely found within cells. During exercise in the absence of sufficient fluid replacement, plasma volume decreases as plasma osmolality and concentration of extracellular fluid Na simultaneously increase, resulting in the onset of hypertonic dehydration (Villiger et al., 2018). The osmolarity data in the current study suggest that postexercise signs of hypertonic dehydration were present at weeks 0 and 5, indicating that even though the dogs had access to water, the intake may not have been enough to offset fluid losses. As these shifts in plasma volume, plasma osmolarity, and extracellular Na occur, fluid is mobilized from the intracellular to the extracellular space in an effort to maintain the extracellular and circulating volume (Nose, Mack, Shi, & Nadel, 1988), suggesting that prolonged exercise may cause greater shifts in intercompartmental fluid and electrolyte movement. Since the acinar cells—the secretory units of the submaxillary gland—facilitate the shift of both water and electrolytes from the plasma into the salivary secretion, the concentrations of salivary electrolytes closely reflect the plasma concentrations (Henriques, 1961; Villiger et al., 2018). As the concentration of Na in the extracellular compartment steadily increases, this theoretically diminishes the necessity for water movement from plasma to the saliva; therefore, alongside the decrease in salivary flow rate, this causes an increase in saliva osmolality as well as protein concentrations (Muñoz et al., 2013; Walsh, Montague, Callow, & Rowlands, 2004). Since changes observed in salivary and plasma osmolality are linked, future research should aim to monitor these parameters simultaneously in response to exercise.

Studies have investigated the exercise‐induced changes to plasma electrolyte concentrations in sled dogs (Ermon et al., 2014; Hinchcliff, Reinhart, Burr, Schreier, et al., 1997); however, these studies were done during multi‐day, extreme distance races (e.g., 300+ mile races) and under in‐field conditions where elements related to diet, water, and supplement intake can be difficult for researchers to control. Ermon et al. (2014) followed teams of Alaskan Huskies during the multi‐day ~1,000‐mile Yukon Quest race and reported mild, yet significant decreases in plasma Na concentrations and unchanged K concentrations. However, approximately 30% of dogs on study were receiving dietary NaCl supplements, a finding that likely contributed to the lack of differences observed (Ermon et al., 2014). In fact, the authors of both studies (Ermon et al., 2014; Hinchcliff, Reinhart, Burr, Schreier, et al., 1997) relied on musher reports for diet and water intakes of the dogs involved, and it is possible that these reports were not accurate. Hinchcliff, Reinhart, Burr, Schreier, et al. (1997)) reported that plasma Na and K concentrations decreased, and Cl concentrations remained unchanged in conditioned Alaskan Huskies following a 70‐hr 300‐mile race. Hyponatremia was reported in response to multi‐day running in cold environments, which contrasts the results of the present study; however, this appears to be related to the timing of blood samples with respect to the ingestion of “watered” foods (Hinchcliff, Reinhart, Burr, & Swenson, 1997). While this suggests that dogs may be able to rehydrate during rests periods—which is supported by Greenleaf et al. (1976), who reported a decrease in the exercise‐induced rise in internal temperature within 5 min of water consumption—it does not address the dehydration that may have occurred during the extended periods of exercise itself. The serum values for the competing dogs also indicate that dehydration may have been present prior to exercising, as prerace concentrations of Na and Cl were either at or above the highest reference values for healthy adult dogs. Hinchcliff, Reinhart, Burr, Schreier, et al. (1997)) report prerace concentrations for Na and Cl of 148.5 and 116 mEq/L, respectively, while the reference range of concentrations for these electrolytes in dogs are 137–149 and 99–110 mEq/L, respectively (Campbell & Chapman, 2000). Ultimately, these reports exemplify the challenges associated with controlling conditions in the multi‐day race and the importance of the timing of blood collections.

In the future, evaluating plasma and salivary electrolyte concentrations simultaneously in dogs subjected to various levels of exercise while being maintained in a controlled environment (e.g., control over diet, food/water intake, exercise) may provide data to support the findings presented herein. These additional data may also help to further our understanding of the fluid and electrolyte shifts that occur between the extracellular and intracellular fluid compartments during exercise. Additionally, since the ability to dissipate heat is hindered by dehydration (Kozlowski et al., 1980; Walsh et al., 2004), researchers should also consider monitoring changes to internal body temperature (e.g., utilizing rectal temperature probes) so as to evaluate how various types or degrees of dehydration and electrolyte imbalance may affect internal temperature during single or repetitive bouts of exercise.

Ultimately, physically active dogs may benefit from an electrolyte supplement prior to, during, and/or after extended bouts of exercise in order to replenish electrolytes lost via exercise‐induced salivation. Furthermore, ingestion of electrolyte‐supplemented fluids prior to a bout of exercise may also assist in the maintenance of lower peak core body temperatures in dogs performing rigorous physical activity, particularly in warmer ambient conditions (Niedermeyer et al., 2020). Additionally, when formulating an electrolyte supplement, other compounds that may aid in mineral absorption and exercise recovery (e.g., glucose, citrate, prebiotics) should be considered aside from just the electrolyte minerals lost during exercise. For example, both glucose and citrate have been reported to increase the absorption of Na (Ermon et al., 2014; Patra, Rahman, Wahed, & Al‐Mahmud, 1990), while inulin‐based prebiotic fibres have been shown to increase solubility of minerals such as Ca and Mg in the gut (Legette et al., 2012; Schuchardt & Hahn, 2017). Finally, the addition of flavorings or palatants to electrolyte‐enriched fluids should be considered as they may play a role in increasing the consumption and/or acceptance by physically active dogs (Otto et al., 2017).

## 5 CONCLUSIONS AND IMPLICATIONS

The data presented herein indicates that salivary concentration of Na, Mg, and Cl increase during exercise, suggesting that these are the electrolytes primarily lost in saliva by exercising dogs. As well, it appears as though changes in the concentrations of select electrolytes (e.g., Cl and Mg) may be related to the duration or intensity of aerobic exercise performed, and as such physically active dogs may benefit from electrolyte repletion alongside basic hydration strategies. While much still remains unknown regarding processes such as how electrolyte mineral compartmentalization may influence secretion of electrolytes into saliva, these data provide the groundwork necessary to move forward in the investigation of production and turnover of canine salivary electrolytes during exercise. Further work is necessary, though, to better our understanding of fluid and electrolyte shifts in exercising dogs and also to evaluate the effectiveness of electrolyte repletion. Follow‐up studies should aim to include a comparative analysis of plasma and salivary electrolyte concentrations and osmolarity, an evaluation of internal temperature in response to various levels of aerobic exercise in dogs, and a quantification of physical conditioning that aligns with the aforementioned parameters.

## DISCLOSURES

M.I.L. works for The Nutraceutical Alliance (Burlington, ON, Canada). J.R.T., N.M., and A.K.S. declare that they have no conflicts of interest. The project was funded by the start‐up money received by A.K.S from the University of Guelph.

## AUTHORS' CONTRIBUTIONS

J.R.T., M.I.L., and A.K.S. designed the research. J.R.T. and N.M. conducted the research, and all authors analyzed the data and wrote the manuscript. A.K.S. had primary responsibility for the final content. All authors read and approved the final manuscript.
